# Difference in muscle synergies of the butterfly technique with and without swimmer’s shoulder

**DOI:** 10.1038/s41598-022-18624-8

**Published:** 2022-09-06

**Authors:** Yuiko Matsuura, Naoto Matsunaga, Hiroshi Akuzawa, Tsuyoshi Kojima, Tomoki Oshikawa, Satoshi Iizuka, Keisuke Okuno, Koji Kaneoka

**Affiliations:** 1 Department of Health and Sports, Niigata University of Health and Welfare, 1398, Shimamicho, Kita-ku, Niigata, Japan; 2 General Education Core Curriculum Division, Seigakuin University, 1-1, Tosaki, Ageo, Saitama, Japan; 3 Department of Physical Therapy, Niigata University of Health and Welfare, 1398, Shimamicho, kita-ku, Niigata, Japan; 4 Faculty of Sport Sciences, Waseda University, 2-579-15, Mikajima, Tokorozawa, Saitama Japan; 5 Department of Sport Sciences, Japan Institute of Sport Sciences, 3-15-1, Nishigaoka Kita-ku, Tokyo, Japan; 6 Yamanashi Gakuin University, 2-4-5, Sakaori, Kofu, Yamanashi, Japan

**Keywords:** Disease prevention, Physiology, Neurophysiology

## Abstract

This study aimed to investigate whether muscle synergy differs between swimmers with and without swimmer's shoulder in the butterfly technique. Muscle synergies, which can assess muscle coordination, were analyzed using surface electromyography. Twenty elite swimmers were included in this study (swimmer's shoulder: n = 8; control: n = 12). The motions involved in executing the butterfly technique were classified into the early pull-through, late pull-through, and recovery phases. Muscle synergy data analyzed using the nonnegative matrix factorization method were compared between the two groups.

The swimming velocities were 1.66 ± 0.09 m・s ^−1^ and 1.69 ± 0.06 m・s ^−1^ for the control and swimmer's shoulder groups, respectively. Four muscle synergies in both groups were identified: synergy #1, which was involved in the early pull; synergy #2, involved in the late pull; synergy #3, involved in the early recovery; and synergy #4, involved in pre- and posthand entry. Compared to the control group, the swimmer's shoulder group had a small contribution from the pectoralis major (*p* = 0.032) and a high contribution from the rectus femoris during the early pull phase (*p* = 0.036). In the late pull phase, the contribution of the lower trapezius muscle in the swimmer's shoulder group was low (*p* = 0.033), while the contribution of the upper trapezius muscle in the pre- and postentry phases was high (*p* = 0.032). In the rehabilitation of athletes with swimmer's shoulder, it is therefore important to introduce targeted muscle rehabilitation in each phase.

## Introduction

Swimmer’s shoulder is the most common injury in swimming^1,2^ since 91% of competitive swimmers experience it in their lifetime^3,4^. In cases of swimmer's shoulder, shoulder pain is particularly frequent and is a major cause of missing practice^5^. Swimming is an overhead sport, highly repetitive upper-limb overhead movements generate most of the propulsion required to execute three major techniques: front crawl, butterfly technique, and backstroke. Elite swimmers swim 14,000 m per day, which requires > 2500 shoulder rotations per day and > 16,000 per week^6^. Swim training volume is associated with shoulder pain in adolescent competitive swimmers^7^. Moreover, in a study assessing the sports injuries sustained during each athletic event at the 2016 Olympic Games held in Rio de Janeiro, swimming was the only sport with a significantly higher incidence of injuries in training than in competition^8^.

Swimmer's shoulder is a painful syndrome of the anterior shoulder induced by repetitive impingement of the rotator cuff beneath the coronal acromion arch^9^. Typically, this diagnosis is labeled "impingement syndrome". Studies on swimmer's shoulder and related factors have frequently been retrospective, with previous research reporting reduced endurance, lack of coordination or weakness of shoulder muscles, lack of scapular stability, poor posture, lack of trunk stability, and changes in shoulder and spinal mobility^9–12^. Repeated stress from shoulder movements in the butterfly and front crawl techniques are thought to cause impingement of the supraspinatus and biceps tendons. In turn, this inflammation can lead to rotator cuff lesions^13^. Factors such as hand positioning and breathing techniques are also known to influence shoulder movements during these events^13–16^. Furthermore, it has been suggested that rotation of the body is related to the etiology of swimmer's shoulder in techniques involving body rotation, such as the crawling and backstroke motions^5,17^. The mechanisms described in these studies also suggest that asymmetric body rotation may contribute to the development of shoulder impingement^5,17^.

In addition to the few studies on shoulder joint movement during swimming, electromyographic studies have been few. In previous studies involving electromyography (EMG) in swimming, Clarys et al. analyzed shoulder and forearm EMGs during front crawl swimming^18^. Figueiredo et al. analyzed the eventual kinematic and electromyographic changes during a maximal 200 m front crawl at race pace^19^. Research on swimmer’s shoulder conducted by means of EMG has focused on the shoulder joint in swimming the butterfly, front crawl, backstroke, and breaststroke^20^. However, considering that previous studies were conducted in the 1990s^21–23^, competition records are rapidly improving now, and the factors of occurrence may be different. Moreover, previous studies have focused only on the surrounding shoulder joint muscles. The swimming motion is based on the trunk and the upper and lower limbs to obtain propulsive power. Therefore, it is possible that not only the muscles around the shoulder joint but also the coordination with trunk and lower limb muscles may affect the development of shoulder joint injuries.

Nonnegative matrix factorization (NMF) analysis^24^, which is based on Bernstein’s concept of muscle synergy, has been used to evaluate muscle coordination. NMF analysis requires EMG data and is divided into two factors: muscle synergy vectors and temporal activation patterns. Muscle synergy vectors indicate muscle coordination, which is a time invariant matrix including the weights corresponding to the contribution of each muscle to the specific, while the temporal activation patterns indicate the activation timing of the muscle synergy^25^. Sports activity has also been analyzed using NMF, although studies have been limited^26^. Studies of NMF analysis have been conducted comparing athletes and nonathletes^27^, as well as before and after training interventions^28^, although only a few studies have examined the presence or absence of injury^29^. Previous studies evaluating coordination during swimming have utilized methods such as movement analysis^30–32^ and muscle synergy analysis^33,34^.

No previous studies have examined muscle synergy differences in the presence or absence of injury. Here, we focused on the butterfly technique, and this study aimed to determine whether muscle coordination of the upper, trunk, and lower limbs during the butterfly technique in elite swimmers differs between those with and without shoulder pain. Because synergies have been reported to vary with injuries and fatigue in other sports, we based our hypothesis on these studies^29,35^. The hypothesis was that swimmers with swimmer’s shoulder would have muscle synergies differing from those in control groups and that swimmers with swimmer’s shoulder would have poor upper limb and trunk/lower limb coordination.

## Materials and methods

### Participants

Twenty young men participated in this study. All participants practiced with a collegiate swimming team 10 times per week. They were elite swimmers; one had received a medal in the butterfly technique in the Rio Olympics, and one had received a gold medal in butterfly swimming in Universiade competitions. Swimmers with a history of lower limb disorders, neurological disorders, or lower limb surgery were excluded. The participants in the control group had experienced no shoulder pain or shoulder injury in their competitive life thus far, and no orthopedic injuries in any part of the body were sustained six months after measurement. The swimmer's shoulder group was defined as those with a history of shoulder joint injury within one year, and the shoulder disorder was confirmed by an orthopedic surgeon. Additionally, the swimmers in the swimmer's shoulder group had no pain at the time of measurement in the electromyographic data but had recurrent shoulder injury within six months of the measurement.

Information about the control group is as follows: n = 12; age, 20 ± 1 years; height, 1.78 ± 0.07 m; weight, 71.3 ± 5.5 kg; International Swimming Federation (FINA) points, 787.1 ± 106.4; and swimming experience, 13 ± 2 years. Details of the swimmer's shoulder group details are as follows: n = 8; age, 21 ± 2 years; height, 1.77 ± 0.06 m; weight, 73.3 ± 6.1 kg; FINA points 756.9 ± 99.1; and swimming experience, 13 ± 4 years. The two groups had no significant differences in characteristics between them. The participants were fully informed of the risks, benefits, and stresses of the study, and their written informed consent was obtained. The study was conducted in accordance with the Declaration of Helsinki and approved by the Waseda University’s Research Ethics Committee (2016–267).

### Experimental setting

Experimental trials were conducted in a 50 m indoor pool. After a 15 min warm up with low- to moderate-intensity aerobic swimming and elements of lower limb movements and drill exercises, the participants performed a 25 m butterfly swimming at the pace of a 100 m butterfly race. They started swimming in the water with a push off from the wall. Two cameras (high-speed camera 1394, DKH Inc., Japan) were installed at 10 m and 15 m from the wall. They filmed the sagittal movements of the swimmers and recorded videos through underwater windows at a 200 Hz sampling rate. Wireless LED markers were placed on the styloid process, lateral epicondyle of the humerus, acromion, and greater trochanter (Kirameki, Nobby Tech, Inc., Tokyo, Japan) to separate the phases of the swimming cycle. The markers were placed on the right side of each participant in the control group and on the injured side of each participant in the swimmer's shoulder group.

### EMG data

**Figure 1:**
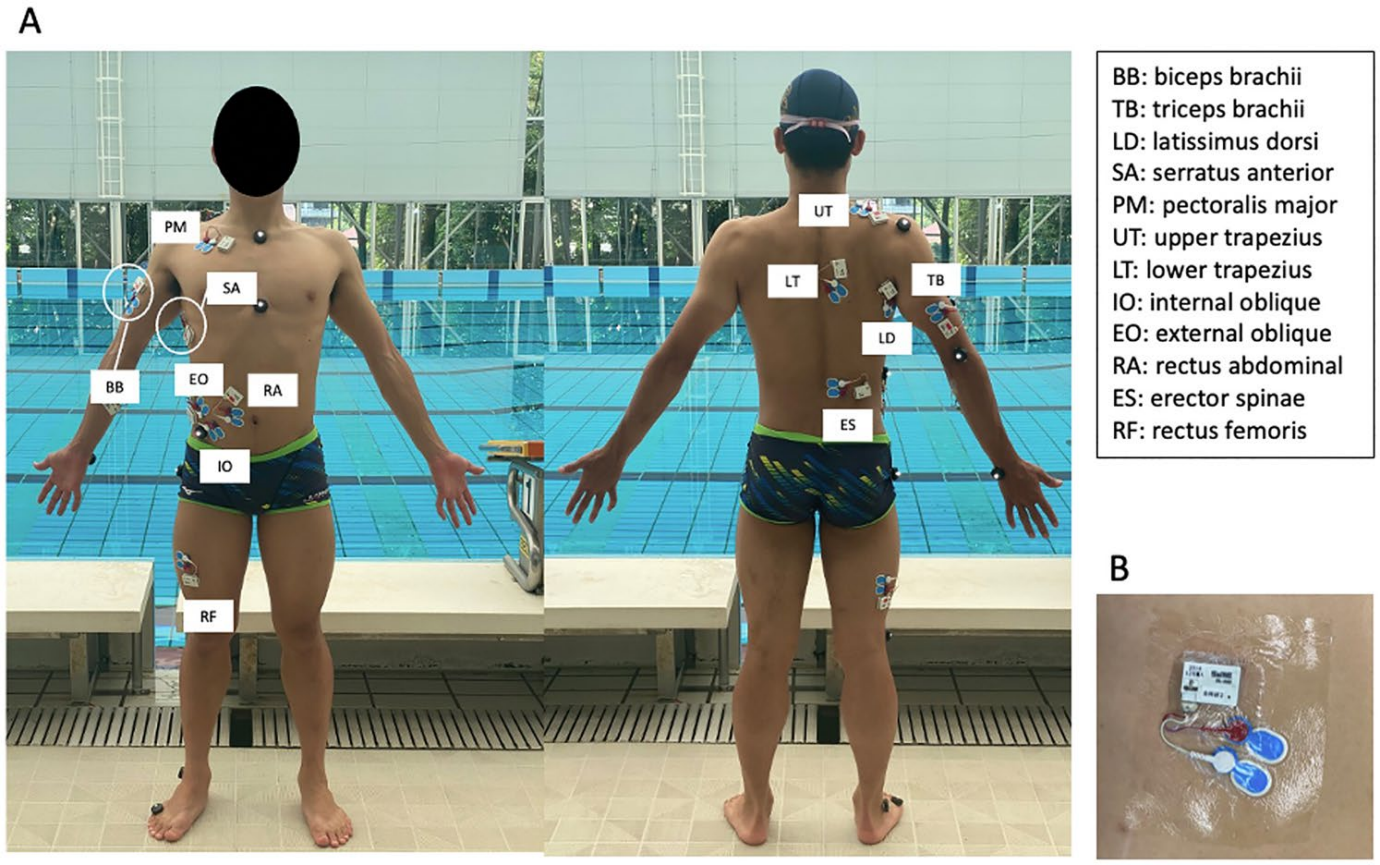
(**A**) Electrode placement. (**B**) The waterproof electrodes were covered with waterproof adhesive sheets to prevent water immersion.

EMG data were measured using a wireless EMG system (Biolog2, S&ME, Inc., Tokyo, Japan), which was recorded from 12 muscles on the right side of the body in the control group and on the injured shoulder side in the swimmer's shoulder group (Fig. 1). Electrodes were placed over the following muscles: biceps brachii (BB), triceps brachii (TB), latissimus dorsi (LD), serratus anterior (SA), pectoralis major (PM), upper trapezius (UT), lower trapezius (LT), internal oblique (IO), external oblique (EO), rectus abdominal (RA), erector spinae (ES), and rectus femoris (RF) (Table 1). Before surface electrode attachment, the skin was rubbed with a skin abrasive and alcohol to reduce skin impedance. The electrodes were attached parallel to the impedance of the muscle fibers to a level < 2 kΩ. Disposable Ag/AgCl surface electrodes were used in pairs (BlueSensor N-00-S, METS Co., Japan). Using the methodology of Figueiredo et al.^19^., the sampling frequency was set at 1000 Hz. The electrodes were waterproofed by covering with a water-resistant tape based on the protocol of Martens et al.^36^. To synchronize the video and EMG data, a synchronizer (PTS-110, DKH Inc., Japan) was connected to both trigger channels. Video and EMG data were recorded simultaneously.

**Table 1:** Muscle abbreviations and electrode placement.

Muscle	Electrode placement
Biceps brachii (BB)	On the line between the medial acromion and the fossa cubit at 1/3 from the fossa cubit^46^
Triceps brachii (TB)	50% on the line between the posterior crista of the acromion and the olecranon at 2 finger widths medial to the line^46^
Latissimus dorsi (LD)	The oblique angle over the LD muscle, approximately 4 cm below the inferior tip of the scapula and midway between the spine and lateral edge of the torso^46^
Serratus anterior (SA)	Vertically placed on the mid-axillary line between ribs 6 and 8^46^
Pectoralis major (PM)	2 cm below the clavicle, precisely medial to the axillary fold^46^
Upper trapezius (UT)	50% on the line from the acromion to the spine on vertebra C7^46^
Lower trapezius (LT)	2/3 on the line from the trigonum spine to the 8th thoracic vertebra^46^
Internal oblique (IO)	Approximately 1 cm medial and inferior to the ASIS^47^
External oblique (EO)	15 cm lateral to the umbilicus^48^
Rectus abdominal (RA)	3 cm lateral to the umbilicus^48^
Erector spinae (ES)	2 finger widths lateral from the spine of L1^48^
Rectus femoris (RF)	50% on the line from the anterior spina iliaca superior to the superior part of the patella on the belly of the muscle corresponding to the central point between the ASIS and upper margin of the patella^46^

### Data analysis

**Figure 2:**
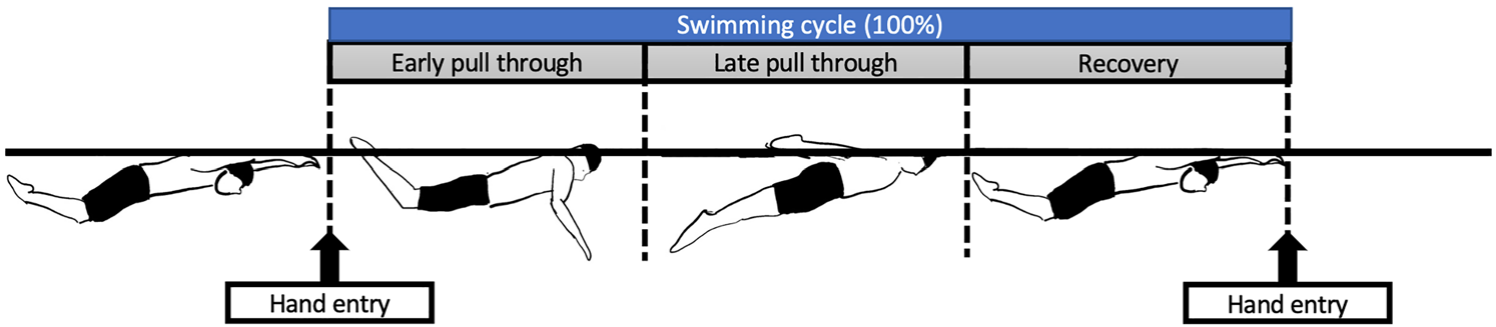
Definitions of the different phases of swimming cycles. (1) Early pull-through started at the beginning of hand entry into the water and ended when the humerus was perpendicular to the axis of the torso connecting the acromion and the greater trochanter. (2) Late pull-through began at the completion of early pull-through and ended as the hand left the water. (3) The recovery phase started at the beginning of the hand exit and ended at hand entry.

Three butterfly technique cycle measurements were analyzed. Each cycle was subject to variations in kinematical parameters^37^. Therefore, three cycle values were analyzed in the present study. Here, one cycle was defined as the time from when the hand first entered the water to when it entered the water again. Assuming one swimming cycle to be 100%, the three phases and the number of data intervals per phase were as follows: The underwater phase with the hand in the water and the recovery phase with the water part were defined for each participant. (1) Early pull-through was defined as the beginning of hand entry into the water and ending when the humerus was perpendicular to the axis of the torso connecting the acromion and the greater trochanter. (2) Late pull-through was the beginning at the completion of early pull-through and ended as the hand left the water. (3) The recovery phase started at the beginning of the hand exit and ended at hand entry. This phase was derived from a report by Pink et al.^38^ (Fig. 2).

The recorded EMG data were analyzed using biomedical information software (BIMUTAS-Video, Kissei Comtec Co., Ltd., Japan). The EMG data were filtered with reference to Vaz et al.^33^. The raw data were bandpass filtered (fourth order Butterworth) between 20 and 450 Hz to reject signal noise. The EMG signals recorded underwater may be affected by artifacts compared to measurements made on land. Therefore, a low-pass filter was used to remove noise and interference in the water. The full wave rectified and linear envelope signals for each muscle were obtained by zero-lag Butterworth low-pass filtering (fourth-order, cutoff frequency of 20 Hz) of the fully rectified EMG signals. EMG data were then normalized relative to each muscle’s associated peak value data and interpolated to 201 time points. Muscle synergy studies often interpolate data to 100 or 101 points; however, since sports movements are quick with multiple changes at a single point, the interpolation of our data was based on a study by Turpin et al., who interpolated muscle synergy data during rowing to 201 points^39^.

### Muscle synergies

A custom MATLAB (MATLAB R2020, MathWorks, Inc., Natick, MA) code was used for the linear envelope and NMF^24^. The EMG matrix (E) was decomposed into spatial muscle synergy vectors (W), which is referred to referred the muscle synergies and their temporal activation patterns (C) by NMF according to Eq. (1) :

1 $${\text{E}} = {\text{WC}} + {\text{e}}$$

2 $$\mathop {\min }\limits_{{\begin{array}{*{20}c} {W > 0} \\ {C > 0} \\ \end{array} }} \left| {\left| {{\text{E}} - {\text{WC}}} \right|} \right|_{{{\text{FRO}}}}$$

E is a p by n initial matrix, “p” is the number of muscles, and “n” is the number of timepoints. The initial matrix consists of the normalized EMG data and consists of the average of three cycles for each of the twelve muscles. E is a 12 by 201 matrix, W represents a p by s matrix, and “s” is the number of synergies and represents muscle synergy. C is an s-by-n matrix that represents the activation coefficient, and “e” is a p-by-n residual error matrix. Eq. (2) shows that matrix “e,” calculated using Eq. (1), is minimized. W is a vector; it is written as $\mathop{W}\limits^{\rightharpoonup}$ when calculated. For each participant, we repeated the analysis by varying the number of synergies between 1 and 11 and selected the least number of synergies fulfilling global variance accounted for (VAF). VAF is defined as 100 × the coefficient of determination from the uncentered Pearson correlation coefficient ^40^. We defined the standard as a VAF > 90%.

VAF was calculated using Eq. (3).

3 $${\text{ VAF}} = \left( {1 - \frac{{\mathop \sum \nolimits_{{{\text{i}} = 1}}^{{\text{p}}} \mathop \sum \nolimits_{{{\text{j}} = 1}}^{{\text{n}}} { }\left( {{\text{e}}_{{{\text{i}},{\text{j}}}} } \right)^{2} }}{{\mathop \sum \nolimits_{{{\text{i}} = 1}}^{{\text{p}}} \mathop \sum \nolimits_{{{\text{j}} = 1}}^{{\text{n}}} { }\left( {{\text{E}}_{{{\text{i}},{\text{j}}}} } \right)^{2} }}} \right) \times 100\;[\% ]$$

where “i” ranges from 1 to “p” and “j” ranges from 1 to “n”. Thus, in this study, “i” was set from 1 to 12, and “j” was set from 1 to 201.

### Statistical analysis

The Shapiro–Wilk normality test with Lilliefors correction was also used to assess the normality of the data for mean swim velocity and the percentage of each phase. Swim velocity was calculated from the time at the 10 m and 15 m passage points. An unpaired t test was performed to compare the mean swim velocity and the percentage of each phase between the groups. Effect sizes were calculated using Cohen’s d for the unpaired t test. The evolution of VAF with the number of synergies extracted was compared between the two populations using repeated measures analysis of variance. We performed an unpaired t test on each of the muscle synergy vectors to compare individual muscle weightings between groups. These comparisons were performed using IBM SPSS Statistics for Windows version 24 (IBM Corp., Armonk, NY, USA). Activation patterns were compared between the two groups using statistical parametric mapping (MATLAB R2020, MathWorks, Inc., Natick, MA). The statistical significance was set at a *p* value < 0.05.

## Results

### Individual data

Table 2 presents the kinematic outcomes during the butterfly technique. Swimming velocities were 1.66 ± 0.09 m・s^−1^ and 1.69 ± 0.06 m・s^−1^ for the control and swimmer's shoulder groups, respectively. The ratios of each phase in one cycle were 35.8 ± 6.1% (control) and 39.2 ± 6.7% (swimmer's shoulder) in the early pull-through phase and 22.5 ± 3.6% (control) and 20.3 ± 3.8% (swimmer's shoulder) in the late pull-through phase. The recovery phase was 41.6 ± 5.0% (control) and 40.5 ± 6.1% (swimmer's shoulder). No significant differences in each phase were observed between the two groups (Table 2).

**Table 2:** Kinematic variables measured during the butterfly technique.

Variables	Unit	Control	Shoulder pain	*p* value	Effect size
Swimming velocity	(m・S^−1^)	1.66 ± 0.09	1.69 ± 0.06	0.44	0.18
Early pull phase	(%)	35.8 ± 6.1	39.2 ± 6.7	0.26	0.27
Late pull phase	(%)	22.5 ± 3.6	20.3 ± 3.8	0.20	0.30
Recovery phase	(%)	41.6 ± 5.0	40.5 ± 6.1	0.66	0.08

The swimming velocity and rate of each phase were not different between the two groups.

### Muscle synergies

**Figure 3:**
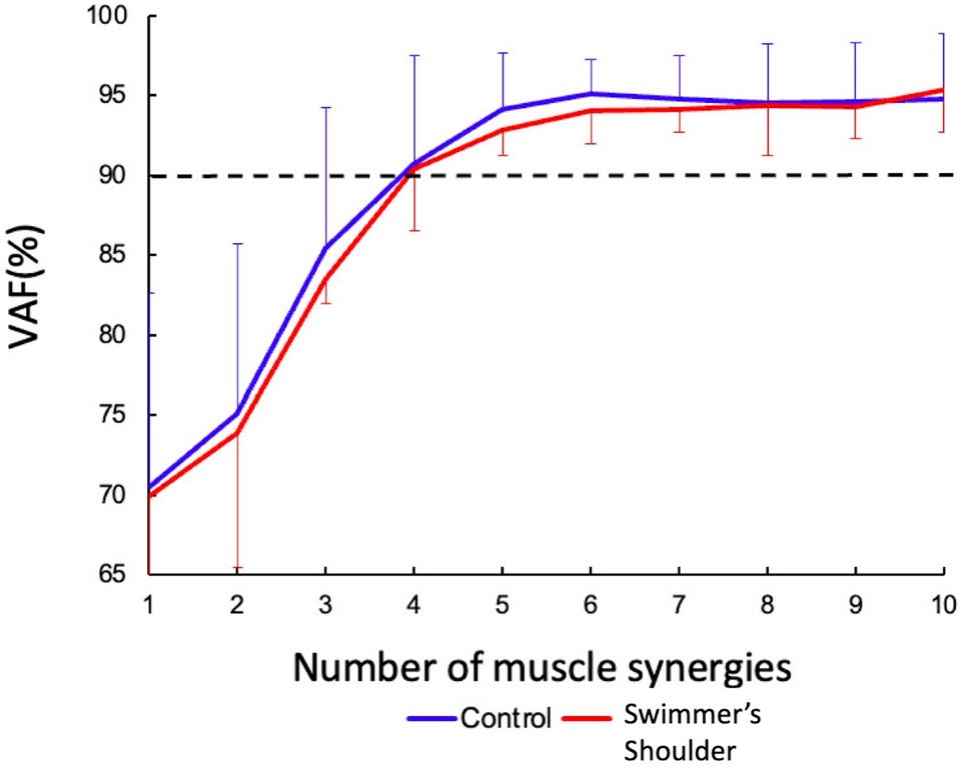
The number of synergies was set as the lowest number for which the global variance accounted for (VAF) exceeded 90%. When the number of synergies was 4, the VAF exceeded 90% in both the control and the swimmer's shoulder groups.

**Figure 4:**
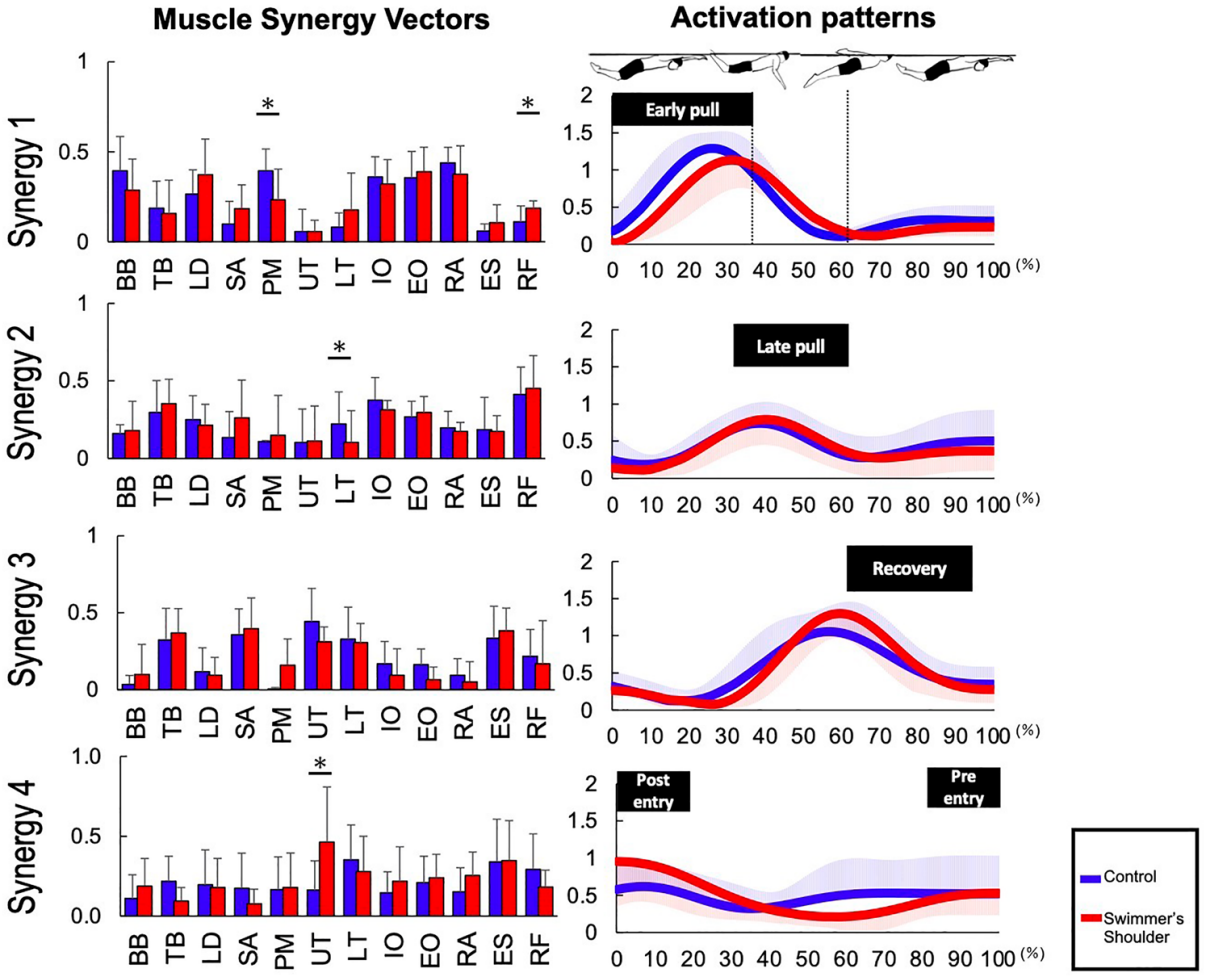
The muscle synergy vectors are shown on the left side of the figure aligned to the corresponding activation coefficient. The synergy activation patterns are shown on the right side of the figure. BB, biceps brachii; TB, triceps brachii; LD, latissimus dorsi; SA, serratus anterior; PM, pectoralis major; UT, upper trapezius; LT, lower trapezius; IO, internal oblique; EO, external oblique; RA, rectus abdominal; ES, erector spinae; RF, rectus femoris.

Figure 3 shows the VAF for each number of muscle synergies in both groups. The extracted number of signals was 4.0 ± 0.85 (mean ± SD) for the control and 4.38 ± 0.52 for the swimmer's shoulder group. There were no significant differences in the values between the conditions (*p* > 0.05). Figure 4 shows four different types of muscle synergies and the corresponding activation patterns (Fig. 4). For each cycle of motion, synergy #1 was active at approximately 20–30% of the cycle, corresponding to the early pull motion; synergy #2 was active at approximately 40%, corresponding to the late pull motion; synergy #3 was active at approximately 55–60%, corresponding to the early recovery motion; and synergy #4 was active at approximately 90–100% and 0–10%, corresponding to actions before and after the hand entered the water.

Synergy #1 is active in the early pull phase, and muscle synergy shows a higher contribution of BB, LD, PM, IO, EO, and RA. The synergy vectors for the PM were 0.40 ± 0.12 for the control and 0.23 ± 0.26 for the swimmer's shoulder groups. The value was larger for the control group than for the swimmer's shoulder group (*p* = 0.032). The synergy vectors for the RF were 0.11 ± 0.09 for the control and 0.19 ± 0.04 for the swimmer's shoulder groups. It was larger for the swimmer's shoulder group than for the control group (*p* = 0.036). Synergy #2 is active in late pull, and the muscle synergy factors are TB, LD, IO, EO, and RF, which have a high contribution. The muscle synergy vector for the LT was larger for the control group than for the swimmer’s shoulder group (*p* = 0.033). Synergy #3 is active in the early recovery, and the muscle synergy shows higher contributions of TB, SA, TU, TL, and ES. These muscle synergy vectors showed no difference between the groups. The activation patterns of Synergy #4 increased before and after the hand entered the water, and the contribution of TU, TL, and ES was high. The muscle synergy vector of the UT for the swimmer's shoulder group was larger than that of the control group (*p* = 0.032). Activation patterns showed no significant difference between the two groups in any of the synergies.

## Discussion

This study is the first to examine the differences in the muscle coordination of the shoulder, trunk and lower limb muscles between swimmers with and without swimmer's shoulder. The number of synergies in each group was four, with synergies active in the early pull (Synergy #1), late pull (Synergy #2), early recovery (Synergy #3), and before and after water entry (Synergy #4). We observed no difference in the timing of the activation patterns of each synergy but found a difference in the muscle synergy vectors.

Synergy #1 is involved in the early pull, and the signals show higher contributions from the BB, LD, PM, IO, EO, and RA. Muscle synergy vectors for the PM and RF differed between both groups (*p* < 0.05) (Fig. 4). The weight of the PM was greater in the control group than in the swimmer's shoulder group (*p* < 0.05). In contrast, the weighting of the RF was greater in the swimmer's shoulder group than in the control group (*p* < 0.05). The early pull phase corresponded to an increase in swimming speed ^37^. The control group was considered to have performed the pull motions using the PM. On the other hand, the swimmer’s shoulder group attempted to use lower limb motions to gain propulsion to compensate for the lack of pull motions using the PM, which may be the reason for the higher RF activity in this group.

Synergy #2 was active at approximately 40%, likely acting with a late pull motion. The contribution of the LT was greater in the control group than in the swimmer's shoulder group. Impingement in the case of swimmer’s shoulder occurs when the scapula is in an elevated position^13^. Since the LT can depress the scapula, we considered that the control group was able to perform the pull movement through depression of the scapula by the LT, thereby avoiding the development of swimmer's shoulder. In addition, in both groups, the muscle weightings of Synergy #2 were high for the TB, LD, IO, EO, and RA; the TB and LD were reported to be active during late pull in previous studies^41^. In addition to these muscles, the IO, EO, and RA were high, and it has been reported that these muscles acted in the execution of undulatory underwater swimming^34^. Therefore, in late pull, the muscle coordination of the TB and LD (involved in the pull movement) and the IO, EO, and RA (involved in the lower limb actions) is important to improve the coordination between the lower limb actions and the pull motion.

Synergy #3 was involved in early recovery, and we observed higher contributions from the TB, SA, UT, LT, and ES in both groups. There were no significant differences in muscle synergy vectors or activation patterns between the two groups. Thus, it is likely that other phases are more involved in the occurrence of swimmer's shoulder due to the butterfly technique. Synergy #4 before and after water entry; at this time, the upper limb is in a flexion/internal rotation position, which is similar to Neer's test position, a shoulder joint impingement test^41^. Excessive activity of the UT and decreased activity of the MT, LT, and SA were observed in individuals with shoulder impingement syndrome^42,43^. In this study, the contribution of the UT in synergy #4 was also higher in the swimmer's shoulder group than in the control group (*p* < 0.05). The involvement of the UT before and after hand entry could therefore induce swimmer’s shoulder. Thus, rehabilitation should include steps to control the contribution of the UT at hand entry.

Integrating the results of this study, we found that the target muscles are different for each phase of the rehabilitation process. Therefore, rehabilitation of butterfly swimmer's shoulder requires phase-specific interventions. Specifically, it is necessary to introduce exercises that increase the contribution of the PM muscle in the early pull phase. In addition, because the generation of lift is necessary to create a propulsive force while swimming^44^, the linkage between the upper and lower limbs is considered particularly important in the early pull phase. It is also advisable to introduce rehabilitative measures that increase the contribution of the LT to late pull, thereby reducing the involvement of the UT pre- and postentry.

This study has limitations that should be noted. A full motion analysis was not performed because of the presence of only two underwater high-speed cameras, and we could not evaluate the 3D motion. Here, motion analysis is used only for phase division and swim speed calculation. Therefore, the definitions of different phases of swimming cycles were also based on previous studies that were conducted in two dimensions^38^, but the butterfly should be divided into down sweep, in sweep, up sweep, and recovery based on a three-dimensional analysis based on more recent previous research^37^. Moreover, swimmers swam 25 m at the 100 m pace rather than their maximum exertion. Since there is no such event as a 25 m swim in competitive racing, measurements were taken at a pace similar to the swimmers' competition pace. Thus, it is possible that they were swimming slightly slower than their actual race pace.

Additionally, because the subjects in this study were high-level participants, the results cannot be generalized to other athlete populations, such as low-level, junior, or master’s swimmers. Martens et al. investigated the electromyography of front crawl swimming and reported that high-level athletes had low intraindividual variability but high interindividual variability^36,45^. Based on these findings, it is necessary to examine interindividual variability by level. Vaz et al. compared the synergies during breaststroke cycles between elite and beginner swimmers and identified three muscle synergies for both beginners and elites. Although the composition was similar between the two populations, the third synergy exhibited high variability within each group^33^. Therefore, the mechanism of shoulder injury occurrence may differ depending on the level of competition and the generation. Moreover, the present study is not a prospective study, so the risk factors cannot be specified. In the future, a prospective cohort study should be introduced, and further investigation is necessary.

## Conclusions and perspectives

A comparison of muscle coordination in the execution of the butterfly technique with and without swimmer's shoulder showed differences in the contribution of muscle synergy in each phase. Subjects with swimmer's shoulder had little involvement of the PM and a high contribution from the RF during the early pull phase; therefore, rehabilitation to maintain the linkage between the upper and lower limb should be introduced. In the late pull phase, the participation of the LT was low, whereas that of the UT muscle in the pre- and postentry phases was high. Therefore, in the rehabilitation of swimmer's shoulder, it is important to introduce targeted muscle rehabilitation in each phase.

## Data Availability

The datasets analyzed during the current study are available from the first or corresponding author on reasonable request.
